# Cognitive deficits in children of alcoholics: At risk before the first sip!

**DOI:** 10.4103/0019-5545.37319

**Published:** 2007

**Authors:** Melvin Chagas Silva, Vivek Benegal, Manjula Devi, C. R. Mukundan

**Affiliations:** Department of Psychiatry, J. N. Medical College, K. L. E. University, Belgaum, India; * Deaddiction Centre, National Institute of Mental Health and Neurosciences, Bangalore, Karnataka, India; ** Department of Psychiatry, National Institute of Mental Health and Neurosciences, Bangalore, Karnataka, India; *** Department of Neuropsychology, National Institute of Mental Health and Neurosciences, Bangalore, Karnataka, India

**Keywords:** Children of alcoholics, externalizing symptoms, P300

## Abstract

**Background:**

High family loading for alcoholism, early onset of alcohol use and childhood disinhibitory behaviors, persisting into adulthood, increase the susceptibility to alcoholism. At the psychophysiology level, reduced amplitude of the P300 component of the Evoked Response Potential is associated with externalizing psychopathology in children. Children of alcoholics have reduced P300 amplitudes. Preliminary data suggests a developmental lag phenomenon in the maturation of the P300.

**Aims:**

The study compares the amplitude and topography of the P300 generated in response to a visual task, between subjects at high risk (HR) and those at low risk (LR) for alcoholism and its relation to externalizing behaviors.

**Results:**

HR subjects have lower P300 amplitudes over frontal brain areas. Differences are greater in young, tending to converge with increasing age. There is a strong association between this reduced brain activation and an excess of externalizing behaviors in HR individuals.

**Conclusion:**

A maturational lag in brain development causing central nervous system disinhibition and externalizing behaviors may underlie the susceptibility to alcoholism.

The alcohol dependence syndrome is a highly heterogeneous construct. Originally conceptualized as a dimensional construct, it has been transformed by recent clinical nosological systems (DSM-IV, ICD-10) into a categorical one for diagnostic purposes.[1]

The clinical construct of alcohol dependence appears to encompass several subtypes of different severity and varying gene environment interactions. One recognizable presentation of alcoholism is a highly heritable form where the disorder aggregates in families and the morbid risk to relatives of alcoholics is significantly higher than the risk to individuals in the general population. Evidence from twin and adoption studies has highlighted the significance of genetic influences, and the heritability of alcoholism has been estimated at 40-60%.[2] The highest risk for developing alcoholism exists for individuals who start using alcohol as adolescents,[3] have a high family loading for alcohol problems and display a cluster of behavioral traits described as disinhibited, under-controlled or impulsive, which are usually evident in childhood and persist into adulthood.[4,5]

It has been suggested that a heritable generalized disinhibitory complex, characterized by a state of central nervous system hyperexcitability, might form the basic diathesis, with each disorder in this spectrum representing a variable expression of this general vulnerability.[4,6]

Aberrant electrophysiological characteristics have been reported in individuals at risk to develop alcohol dependence, as well as related disinhibitory disorders. These include a range of neuro-electric measures of (a) spontaneous brain electrical activity, such as the increased beta power in the resting electroencephalogram;[7] and (b) neurocognitive markers of attentional processing, such as the reduced amplitude of the P300 component of the event-related potential (ERP).[8] These are hypothesized to reflect a state of central nervous system (CNS) hyperexcitability resulting from an inherited homeostatic imbalance between the excitatory and inhibitory brain neurons, which represents a central vulnerability factor for developing alcohol dependence.[9]

Among the electroencephalographic measures used in studies of both substance-dependent patients and individuals at risk for substance dependence is the P300 component of the ERP. P300 denotes a wave of brain electrical activity emerging approximately 300 msec following the presentation of a rare or surprising stimulus - particularly when the occurrence of the stimulus must be actively acknowledged by the subject, e.g., by pressing a response key. P300, therefore, appears to reflect a cognitive operation associated with revising the mental representation of attended aspects of the recent or current environment.[10]

The P3 component of the ERP, irrespective of the task and modality, is associated with an inhibition of neuron assemblies involved in perceptual processing of the attended sensory input, thereby achieving a “closure” of the cognitive operations dealing with the currently attended sensory input.[11,12] The amplitude of the P300 thus indexes brain actions involved in “the maintenance of working memory”[13] and is proportional to the amount of attentional resources employed in a given task.[14] There is ample evidence that ERP waveforms are highly heritable.[15,16]

The association of reduced P3 amplitudes with high sensation-seeking, particularly with high disinhibition, has been identified in adult children of alcoholic subjects,[17] and low P3 amplitude is associated with externalizing psychopathology in adolescents.[18–20] Thus, a low P3 amplitude would indicate a state of disinhibition.[9,11]

There is a long line of evidence for considering the P3 amplitude of the ERP as an endophenotype for the risk of alcoholism.[9] Starting with the original findings of Begleiter *et al.*[21] of reduced P3 amplitudes in the sons of alcoholic fathers, who had no prior exposure to alcohol, multiple studies have demonstrated that the P300 amplitude is smaller in high-risk child/adolescent offspring of alcoholics (HR) than in controls.[9] Benegal *et al.*[22] reported that children of Early-Onset Alcoholics (those who had developed alcoholism before the age of 25 years) had relatively lower P3 amplitudes than children of Late Onset Alcoholics and children of non-alcoholics.

A meta-analysis of 22 P300 studies[23] has shown that while P300 was reduced overall in HR compared to low-risk offspring (LR), the differences were more pronounced in younger subjects, with few differences being seen in young adulthood. Preliminary evidence gathered from the comparison of developmental trajectories of P300 amplitude between HR and LR groups also shows that HR subjects appear to be delayed in reaching age-appropriate P300 amplitude compared to controls.[24]

The evidence from the available literature suggests that the susceptibility for developing alcoholism (especially the early-onset subtype) may be mediated by a state of altered CNS functioning which can be quantified in terms of measurable cognitive deficits (P300 amplitude). There is speculation that these deficits may be consequent to delays in brain development and that they may be proximately associated with a cluster of disinhibited, under-controlled or impulsive behavioral traits usually evident in this high-risk population from childhood and persisting into adulthood.

The current study aimed to explore differences in cognitive function (visual P300 amplitude), including topographic variations if any, between subjects at high risk for alcoholism (HR) and those without such risk (subjects at low risk for alcoholism; LR). The study also aimed to investigate the link between deficits in cognitive function (P300 amplitude) and the manifest behaviors (externalizing spectrum) across the groups. A subsidiary aim was to examine the function of age (and by extension, the developmental stage of the CNS) with the magnitude of cognitive deficit, if any.

We hypothesized that the HR group would have smaller visual P300 amplitudes than the LR group; that there would be a strong association between reduced P300 amplitude and externalizing symptoms; and that the largest differences between HR and LR groups would be seen in the younger subjects rather than in the older subjects.

## MATERIALS AND METHODS

### Subjects and controls

Twenty-five “high risk” (HR) male offspring (age range of 5-25 years) of treatment-seeking patients with alcohol dependence [Diagnostic and Statistical Manual of Mental Disorders - Fourth Edition (DSM-IV)][25] and 25 “low risk” control (LR) subjects (age- and sex-matched offspring of volunteers) were recruited for the study. “High risk” (HR) in this study was defined to denote alcohol-naïve offspring of early-onset (having developed dependence before 25 years of age) alcohol-dependent fathers with two or more alcohol-dependent first-degree relatives. “Low risk” (LR) was defined as alcohol-naïve individuals with absence of family history of alcohol dependence in any of the first- or second-degree relatives.

The age range was decided in order to cover the developmental span of the visual P300.

### Interview procedure

The Semi-Structured Assessment for Genetics of Alcoholism-II (SSAGA-II), adult version,[26] was used to rule out any lifetime psychiatric disorder (other than alcohol dependence) in fathers of HR subjects and to rule out any lifetime psychiatric diagnosis (including alcohol dependence) in both parents of LR and in mothers of HR subjects. The family history of psychiatric disorders in first-degree relatives of both HR and LR was gathered using the Family Interview for Genetic Studies (FIGS, NIMH Clinical Neuro-genetic Initiative)[27] from three or more adult informants in the family. None of the HR had family history of psychiatric disorder (other than alcohol dependence) in their first-degree relatives. None of the LR subjects had family history of psychiatric disorder (psychoses, anxiety disorder, affective disorder, obsessive-compulsive disorder or Tourette's syndrome, including alcohol dependence) in their first-degree relatives.

The subjects were then assessed using the SSAGA-II (child, adolescent or adult versions as indicated) for externalizing symptoms (attention deficit, hyperactivity, impulsivity, conduct and oppositional-defiant symptoms) and to rule out any other syndromal psychiatric diagnoses (psychoses, anxiety disorder, affective disorder, obsessive-compulsive disorder or Tourette's syndrome). The SSAGA items pertaining to inattention, hyperactivity, impulsivity, oppositional-defiance and conduct symptoms were added to calculate the respective subscales and summated to derive a total externalizing symptoms score (ESS).

An experienced neurologist ruled out any neurological disorder, mental retardation or history of significant head injury, as these were specific exclusion criteria. All subjects were right-handed as assessed by Annett's questionnaire.[28]

The Institute Ethics Committee approved the study. After providing complete description of the study to the subjects, written informed consent was obtained from all participants and parents of subjects under 18 years of age. The procedure was conducted in accordance with the declaration of Helsinki.

### Evoked response potential recording

Visual evoked response potentials were recorded during 250 trials with simple target/nontarget visual task. The ratio of target to nontarget was 1:4. The paradigm involved responding to randomly generated stimuli consisting of a rectangle whose angle of presentation varied between horizontal and vertical. The subject had to respond only to the target stimulus - in this case, the vertical rectangle. The ERP was recorded on an EEGSYS machine. This is a computer-based “electrophysiological data”-gathering and analysis system consisting of electrodes fixed on the head via an electrode cap. The analog signals with reference to linked electrodes are sent to the head box and then to a computer circuit board for further amplification, frequency filtering and conversion to digital values. The signal data was then saved as digital data. The electrode positions were frontal (AF3, AF4, Fp1, Fp2, F1, F2, F3, F4, F5, F6, F7, F8, FCz and Fz), central (C1, C2, C3, C4, C5, C6 and Cz), parietal (P3, Pz and P4), temporal (T1, T2, T3, T4, T5 and T6) and occipital (O1 and O2). Vertical eye movements were recorded from electrodes placed above and below the right eye, and horizontal eye movements were recorded from electrodes placed at the outer canthus of each eye. A lead placed above the center of the forehead served as the ground electrode. Electrode impedance was below 5 kΩ. Recordings were done using a Biolink amplifier system with a low-pass filter of 100 Hz (for EEG) and 30 Hz (for ERP) and high-pass filter of 0.3 Hz (for EEG) and 0.01 (for ERP), and signals were amplified up to 15,000 times. Calibration was done with a 4-8-Hz sine wave with amplitude of 50 mV generated from a Nihon-Kohden signal generator. For the ERP measures, a 50-Hz notch filter was used for the frequency rejection.

### Analysis

The collected data was coded and entered into a statistical package (Statistical Package for Social Sciences, Version 11.0). Data distributions were examined for normality and analyzed using descriptive statistics such as frequencies, means and standard deviation. Since the data did not satisfy normal distribution even after log transformation, we used nonparametric tests to look for between-group differences. Accordingly, the Mann-Whitney *U*-test was applied to look at between-group differences. Bivariate correlation analysis using the Pearson's correlation coefficient was used to find out significant correlations between the P300 amplitudes and other clinical variables. Finally, head-to-head comparison between age-matched subjects and controls was attempted using Scatter Overlay plots to look at the changes in the P300 amplitude over different ages.

## RESULTS

### Sample description

The final data analyses were performed on data from 24 HR and 25 LR subjects. One HR subject had to be excluded from the analysis as the EEG data was corrupted. The ages of the children ranged from 6 to 25 years, with a mean of 14.3 ± 4.8 years.

The fathers of the HR group all satisfied DSM-IV criteria for alcohol dependence and had developed dependence by 19.95 (2.54) years of age and had on an average three other affected first-degree relatives with alcohol dependence (minimum three, maximum eight). They drank 17.3 (8) standard drinks (12 g ethanol) daily or almost daily. Neither the mothers of the HR group nor both parents of the LR group satisfied criteria for a diagnosis of lifetime alcohol dependence.

### Differences in externalizing symptom scores between HR and LR subjects

The HR subjects had significantly higher scores than the LR subjects on hyperactivity, impulsivity, inattention, total attention-deficit hyperactivity disorder (ADHD), conduct, oppositional defiant disorder (ODD) and total externalizing symptoms score (ESS) [Table 1].

**Table 1:** Externalizing symptom scores in high risk and low rish subjects

Clinical variables	High rish	Low risk	*t* df 47
Attention deficit hyperactivity disorder			
Inattention	4.8 ± 3.1 (0-9)	1.2 ± 0.9 (0-3)	5.6*
Subscores			
Impulsivity	1.6 ± 1.1 (0-3)	0.4 ± 0.2 (0-1)	7.8*
Hyperactivity	3.6 ± 1.9(0-6)	0.5 ± 0.7(0-2)	6.7*
Total	10.0 ± 5.3 (0-17)	1.7 ± 1.3(0-4)	7.3*
Conduct subscores	2.1 ± 2.8(0-11)	0	3.8*
Oppositional defiant disorder subscores	2.7 ± 2.6 (0-7)	0.6 ± 0.3 (0-1)	5*
Externalizing symptoms (total scores)	14.8 ± 9.2 (0-31)	1.8 ± 1.3 (0-4)	47*

* *P* <0.0001

### Differences between groups on electrophysiological data

Independent groups *t*-test analysis (Mann-Whitney *U*-test) [Table 2] revealed significantly lower P300 amplitudes over the frontal leads, namely, AF4 (*P* = 0.03) and F3 (*P* = 0.04) groups, in the HR group compared to the LR group. P300 amplitudes at AF3 (*P* = 0.07) and F4 (*P* = 0.06) electrodes were also lower in the HR group, but the differences did not reach statistical significance.

**Table 2:** P3 amplitudes at various electrode positions in high risk and low risk groups: mean, standard deviation, significance

Electrode	HR group		LR group		P*
			
	Mean	S.D.	Mean	S.D.	
AF3	-10.2	19.5	-2.3	11.0	0.07
AF4	-11.62	21.3	1.5	46.9	0.03
F5	3.6	27.8	-3.7	14.9	0.70
F3	-10.9	28.5	0.7	11.7	0.04
F1	1.8	31.6	-5.1	14.9	0.73
FZ	6.1	56.7	-0.9	10.6	0.30
F2	6.4	59.2	-8.0	16.7	0.97
F4	1.2	30.7	0.51	6.8	0.06
F6	1.6	36.6	-2.9	13.7	0.73
FCZ	4.2	37.8	-1.7	12.4	0.75
C5	-0.15	21.2	0.8	10.0	0.75
C3	10.4	42.6	1.7	10.9	0.75
C1	17.5	71.6	5.6	16.3	0.75
CZ	12.1	51.6	1.5	12.5	0.97
C2	13.6	54.4	1.8	9.0	0.70
C4	16.7	78.0	-5.8	54.2	0.70
C6	9.27	50.9	-9.2	53.3	0.94

* Mann-Whitney *U*-test

### Overlay scatter plots of the developmental trends in P300 amplitude

**Figure 1:**
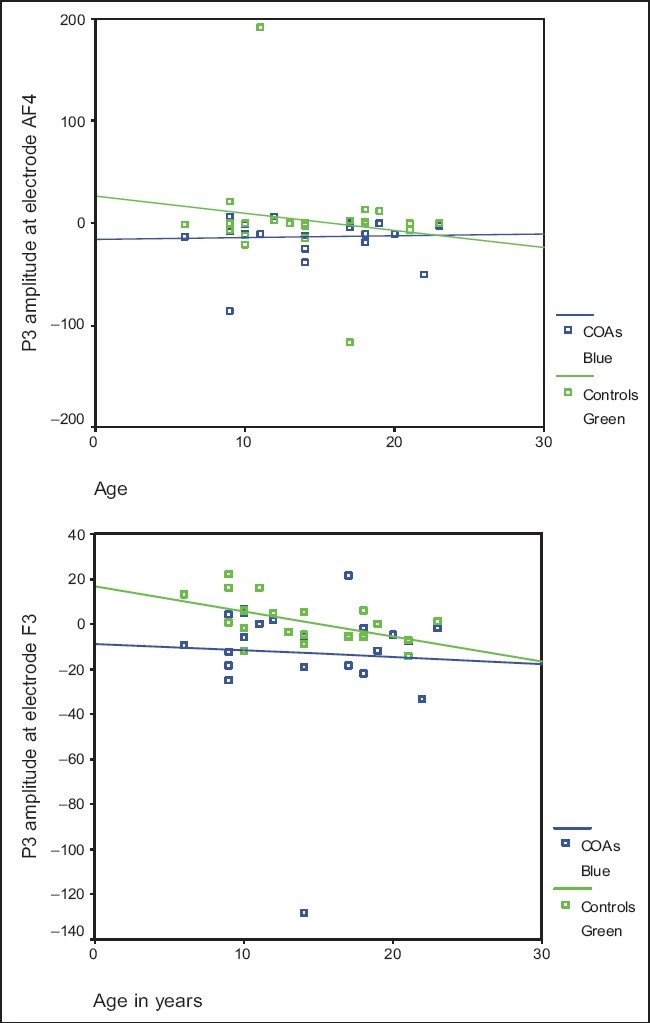
Shows overlay scatter plots for P3 amplitude in HR (blue) and LR (green) over various ages at electrode AF4 and F3

Scatter plots were charted with the P300 amplitudes of HR and LR subjects on the y-axis and ages of subjects on the x-axis. The best-fit line for the scatter for HR and LR subjects was developed. These charts were plotted only for the frontal electrodes which had shown to be significantly different in the across-groups comparison using the Mann-Whitney *U*-test. The graphs for the P300 amplitudes at AF4 and F3 show that in LR subjects, the P300 amplitude is high in the younger age groups and tends to decrease with age. In the HR subjects, however, the P300 amplitude is significantly lower than in controls in the younger age groups but tends to increase with increasing ages, converging or tending to converge at around age 20 years [Figure 1].

### Correlations between clinical and electrophysiological variables

ADHD impulsivity subscore was negatively correlated with the P3 amplitude at the frontal electrode F3 at adequate levels of significance (Pearson's correlation coefficient = -0.33; *P* < 0.02). Similarly the same subscore tended to correlate significantly with the P3 amplitude at another frontal electrode, AF3 (Pearson's correlation coefficient = -0.263; *P* < 0.048,). Correlations with other externalizing symptom profiles did not reach significant levels.

## DISCUSSION

The present study demonstrated that subjects at high risk for alcoholism (child/adolescent/young adult offspring of early-onset alcoholics with high family loading for alcoholism) have deficient cognitive processing capabilities compared to low-risk control subjects, as indexed by significantly lower visual P300 amplitudes.

This deficit appears to be significantly associated with a range of externalizing behaviors which also differentiate these HR subjects from the LR controls and appears to be localized to the frontal regions of the brain. The differences in P300 amplitude are more prominent in the youngest subjects, with the trajectories in HR and LR groups tending to converge with adulthood. This may be indicative of a developmental delay or deficit.

The lower P300 amplitude observed in the male child/adolescent/young adult offspring of early-onset alcoholics with strong family loading for alcoholism, in this study, replicates earlier findings which have documented similar cognitive deficits which appear to consistently differentiate HR from LR subjects.[9] These deficits in cognitive processing are present long before exposure to alcohol and may therefore be presumed to represent a mediating factor in the susceptibility to early alcohol-related problems in this population.

The lower P300 amplitude is thought to index a state of central nervous system (CNS) disinhibition.[9,11,17–20] A recent study from our group, studying the hyper-polarization response in the CNS, consequent to the depolarization caused by transcranial magnetic stimulation, has documented that HR subjects have significantly lower capacity for cortico-cortical and trans-cortical inhibition relative to LR subjects.[29]

The strong association of lowered P300 amplitude and lowered inhibitory capacity in the CNS[29] with the spectrum of externalizing/disruptive behaviors observed in the HR group fuels speculation that the observed P300 deficits in the HR group are an index of CNS disinhibition, which is manifest behaviorally as a spectrum of externalizing or disinhibitory/disruptive behaviors. This reduction in P3 amplitude is not only observed in alcoholism but also in a spectrum of disinhibitory disorders, such as conduct disorder (CD), attention-deficit hyperactivity disorder (ADHD), oppositional defiant disorder (ODD) and antisocial personality disorder (ASPD).[11,30]

It has been proposed that behavioral phenomena such as psychopathy, antisocial and impulsive traits and alcoholism should be viewed as variable expression of a generalized disinhibitory complex.[31] Substance dependence, such as alcohol dependence, has been considered part of the disinhibitory/externalizing disorder spectrum,[32] as these disorders coexist in their clinical presentation; they co-occur with externalizing traits in both children and adults[33] and share similar electrophysiological indices such as a reduced P300 amplitude.[34] Clinically, greater impulsiveness (action without planning or behavior that is prematurely executed and has maladaptive consequences) is one of the most common manifestations of these disinhibitory disorders[35,36] and appears to be associated with a failure of behavioral filtering processes outside of consciousness and with compromised capacity to use appropriate judgment to reflect on impending acts. The prevalence of increased impulsivity and other externalizing symptoms among substance abusers has been consistently documented. Alcoholic subjects have higher levels of impulsivity, particularly those with early-onset-type alcoholism.[37]

A community-based twin study found a strong association of reduced P300 amplitude with attention deficit hyperactivity disorder, oppositional defiant disorder, conduct disorder, antisocial personality disorder and alcoholism. Reduced P3 amplitude at age 17 predicted the development of substance-use disorders at age 20.[38]

We have also documented that the maximum differences in P300 amplitude between HR and LR subjects is maximal in the younger children and that the trajectories of amplitude tend to converge with increasing age. This supports earlier findings that have recorded that HR subjects appear to be delayed in reaching age-appropriate P300 amplitude compared to controls. This is highly suggestive of developmental delays in cognitive development in the HR subjects relative to their LR peers. Production of P300 is believed to be involved in inhibition of brain activities: the larger the P3 amplitude, the more the neurophysiological inhibition.[17–20] Therefore, a low P300 amplitude in alcoholics is suggestive of lack of inhibition, i.e., neurophysiological disinhibition. In the present study, the findings of increased disinhibitory behaviors in HR subjects and significant negative correlations between visual P300 amplitudes and externalizing symptoms provide evidence that links a basic neuro-electric endophenotypic marker to a multidimensional behavioral construct, impulsivity.

It must be acknowledged that the best paradigm for tracking developmental delay is to perform periodic longitudinal evaluations rather than the cross-sectional measurement across a developmental age span, as adopted in this study. Due to logistic difficulties, such a longitudinal assessment was not possible. Nevertheless, the growth trajectories of the P300 amplitude observed in our study closely resemble those observed in the single study using a longitudinal design.[8] Similar to the growth curves obtained in the longitudinal design, these children/adolescents/young adults (ages 5-25) showed a steady decline for visual P300 (auditory P300 shows an increase) with a consistent trend for the HR subjects to be delayed in reaching age-appropriate P300 levels when compared to controls.

We recruited only male offspring as the heritable form of alcoholism is acknowledged to be “male limited.”[39] However, future studies which include female offspring will increase the scope of these findings.

The differences in P300 amplitude between HR and LR subjects in this study were most prominently observed in the frontal areas of the brain.

A recent study showed that early-onset alcoholic subjects manifested reductions in target P3 amplitudes and that the reduced activation was mapped in the cingulate, medial and superior frontal regions in alcoholic subjects and highly impulsive subjects.[40] Positron emission tomography (PET) and functional magnetic resonance imaging (FMRI) studies have demonstrated similar results of decreased activities in the frontal lobe in the high-risk offspring of alcoholic subjects.[7] These findings strongly suggest that the frontal lobe and the circuitry connections to the limbic structures are vulnerable substrates in alcoholic subjects, even before they have began drinking, and this may be one of the indicators of a predisposition to developing alcoholism.

Localization studies employing both functional magnetic resonance imaging and dipole modeling techniques suggest that the P300 recorded from the scalp originates from neural generators in frontal (anterior cingulate) and parietotemporal (supramarginal gyrus) regions of the brain.[41] An earlier study reported that boys with externalizing symptoms (a history of rules violations) failed to exhibit the normal maturational increase in P300 amplitude found in boys without a history of rules violations. Topographic analyses of current source densities suggested that the source of the maturational deficit involved P300 generators within the frontal brain. Parietal generators of P300 matured normally. Adolescents with externalizing disorders fail to show the same maturational increase in frontal P300 found in normal adolescents. In contrast, P300 measured in posterior brain regions increased equally with age in adolescents with or without externalizing disorders.[42]

Our group has recently reported that male child/adolescent/young adult offspring of early-onset alcoholics with strong family loading for alcoholism (HR) have smaller gray matter volume in frontal and cingulate gyri, amygdala, hippocampus, parahippocampus, thalamus and cerebellum in comparison to LR control subjects. Also, these deficits significantly predicted the excess of externalizing spectrum behaviors in HR individuals. Significantly, the differences in volume between high- and low-risk subjects are also found to be maximal in younger subjects and tend to decrease among the older subjects. We have interpreted these findings of differences in developmental trajectories of brain growth between the two groups to represent a process of maturational lag, affecting regions known to be actively developing during adolescence and critical to the neurocircuitry of motivation and reward.[43] It is significant in this context that the age-related changes in P300 amplitude are found only in frontal brain regions. It is well accepted that the frontal brain regions (cingulate gyrus, prefrontal lobe, etc.) increase in volume and continue to mature during childhood and adolescence.[41–43] The developmental changes seen in the growth trajectories of P300 amplitudes during adolescence, in all likelihood, appear to be controlled by still maturing frontal generators, with the contribution from the parietal generators remaining constant. In the HR offspring of alcoholics, the delayed maturation of these areas of the brain, as observed, may lead to a lag in the production of age-appropriate P300 amplitudes, due to an inadequacy of the inhibitory mechanisms of the CNS. This in turn, we hypothesize, is likely to be manifest as an excess of externalizing/disruptive behaviors. In the broader context, these results are consistent with evolving theories of ADHD and other disruptive behavior disorders. Patients with ADHD have been documented as exhibiting smaller changes in brain structure[44,45] and function[46] during adolescence.

The greater impulsivity predisposes these individuals to early experimentation with substances of abuse, as well as engages them in a variety of high-risk activities. We have also previously documented that HR individuals (as defined in this study) also experience greater reinforcing effects than LR individuals, from an equivalent dose of ethanol. Spectral analysis of the electroencephalogram (EEG) showed distinct patterns of response to ethanol in the two groups. The HR individuals showed early and prolonged relaxing effects (increased alpha 1 activity), while the LR group showed an initial relaxing effect, which appeared slower than in the HR individuals; followed by a relatively early, prolonged activation effect (increased beta activity). Significantly, the HR group, in contradistinction to their heightened CNS response, appeared to have a lower subjective response (feeling less drunk) than the LR individuals. This differential response in these individuals is then likely to result in early, repeated alcohol use, thus increasing the risk of developing early-onset alcohol dependence.[47]

Thus, in this HR population, the processes which underlie the risk of developing alcohol dependence set in long before the first sip of alcohol by the subjects of this population.

In this study we have demonstrated reduced activation, during processing of visual targets, of sources in the frontal lobes in individuals with a high susceptibility to alcoholism. The growth trajectory of this measure across the developmental lifespan from childhood to young adulthood leads us to hypothesize that these deficits may be due to a lag in brain developmental processes in these subjects. We have also found a strong association between this reduced brain activation and an excess of externalizing behaviors in these individuals. Our data thus provide evidence of a link between an index of neuronal disinhibition and a manifestation behavior, namely, externalizing symptoms, which are thought to be an important factor that underlies the pathogenesis of alcohol dependence.
